# Understanding the experience of initiating community-based group physical activity by people with serious mental illness: A systematic review using a meta-ethnographic approach

**DOI:** 10.1192/j.eurpsy.2020.93

**Published:** 2020-10-22

**Authors:** Helen Quirk, Emma Hock, Deborah Harrop, Helen Crank, Emily Peckham, Gemma Traviss-Turner, Katarzyna Machaczek, Brendon Stubbs, Michelle Horspool, Scott Weich, Robert Copeland

**Affiliations:** 1 School of Health and Related Research (ScHARR), University of Sheffield, Regent Court, Sheffield, United Kingdom; 2 Health and Social Care Research, Sheffield Hallam University, Sheffield, United Kingdom; 3 Department of Health Sciences, University of York, Heslington, United Kingdom; 4 Leeds Institute of Health Sciences, University of Leeds, Leeds, United Kingdom; 5 Advanced Wellbeing Research Centre, Sheffield Hallam University, Sheffield, United Kingdom; 6 Department of Psychosis Studies, Institute of Psychiatry, Psychology and Neuroscience (IoPPN), King’s College London, London, United Kingdom; 7 Sheffield Health & Social Care NHS Foundation Trust, Sheffield, United Kingdom

**Keywords:** Adults, initiation, meta-ethnography, physical activity, serious mental illness

## Abstract

**Background:**

People living with serious mental illness (SMI) experience debilitating symptoms that worsen their physical health and quality of life. Regular physical activity (PA) may bring symptomatic improvements and enhance wellbeing. When undertaken in community-based group settings, PA may yield additional benefits such as reduced isolation. Initiating PA can be difficult for people with SMI, so PA engagement is commonly low. Designing acceptable and effective PA programs requires a better understanding of the lived experiences of PA initiation among people with SMI.

**Methods:**

This systematic review of qualitative studies used the meta-ethnography approach by Noblit and Hare (1988). Electronic databases were searched from inception to November 2017. Eligible studies used qualitative methodology; involved adults (≥18 years) with schizophrenia, bipolar affective disorder, major depressive disorder, or psychosis; reported community-based group PA; and captured the experience of PA initiation, including key features of social support. Study selection and quality assessment were performed by four reviewers.

**Results:**

Sixteen studies were included in the review. We identified a “journey” that depicted a long sequence of phases involved in initiating PA. The journey demonstrated the thought processes, expectations, barriers, and support needs of people with SMI. In particular, social support from a trusted source played an important role in getting people to the activity, both physically and emotionally.

**Discussion:**

The journey illustrated that initiation of PA for people with SMI is a long complex transition. This complex process needs to be understood before ongoing participation in PA can be addressed. Registration—The review was registered on the International Prospective Register of Systematic Reviews (PROSPERO) on 22/03/2017 (registration number CRD42017059948).

## Introduction

Individuals living with serious mental illness (SMI), inclusive of major depressive disorder, schizophrenia and bipolar disorder, experience premature mortality [1], increased morbidity (e.g., type 2 diabetes [2], metabolic syndrome, and cardiovascular disease [3]), and higher rates of obesity [4,5] are compared with the general population.

Reducing the premature death rate by targeting the physical health conditions experienced by people living with SMI is complex and multifactorial. One way to address this mortality gap is by modifying behavioral risk factors [6–8], including physical inactivity (or sedentary behavior). Physical activity (PA), encompassing the wider domains of exercise and sport, may have a crucial role in addressing the health inequalities experienced by people living with SMI, in addressing premature mortality, in preventing the onset of comorbidities, and in improving the overall health and wellbeing of this population [9].

The benefits of PA for people living with SMI include improvements in psychiatric symptoms, quality of life, physical fitness, cardiometabolic risk factors, body mass index, and weight [10]. There is also promising evidence that community-based PA (in a group situation) can reduce social isolation, stigmatization, and can enhance social identity in people living with SMI [11–14]. Indeed, the World Health Organization’s *Mental Health Action Plan 2013–2020* called for the provision of mental health services integrated in communities for service users and families [15]. People living with SMI, however, engage in significantly less PA and greater amounts of sedentary behavior compared with the general population [6–8,16,17]. For example, half of the people living with SMI do not meet the guidelines of 150 min per week of moderate intensity PA [17]. This is despite research showing that people living with SMI want to undertake PA [18,19].

A better understanding of how to promote PA in a way that is engaging, appealing, and socially supportive for people living with SMI is much needed. With this in mind, the purpose of this review is to explore the *initiation* of community-based group PA in people with SMI. We define initiation as “the period in which people start being more physically active (also referred to as “adoption” and “uptake”)” (p. 3) [20]. Given the range of barriers faced by people living with SMI when trying to engage in PA (e.g., low mood, stress, or lack of support) [19], a thorough exploration of the lived experience of initiation, rather than maintenance of PA, appears important. To achieve this, we undertook a meta-synthesis [21] of qualitative studies adopting a meta-ethnographic approach [22]. Meta-ethnography seeks to uncover a new understanding of a phenomenon that is greater than that contained within individual studies. Its strength lies in its attempt to preserve the interpretive properties of the original qualitative data. Specifically, our meta-ethnography aimed to:

- Systematically search and appraise qualitative research on the experience of initiating community-based group PA for adults living with SMI.
- Synthesize findings from existing research regarding the experience of initiating community-based group PA and key features of social support within these contexts for people living with SMI.
- Identify from participants’ experiences the active ingredients that could inform future interventions to improve uptake of community-based group PA among people living with SMI.

## Methods

Detailed information on the methods undertaken in this review are published in the protocol [20]. The review was registered in the International Prospective Register of Systematic Reviews: CRD42017059948. Noblit and Hare’s (1988) meta-ethnography approach comprises seven stages, with the review authors moving back and forth between stages four and six as ideas for translation and synthesis are explored. The review is reported in accordance with eMERGe meta-ethnography reporting guidance [23]. The findings of the review were discussed with two individuals living with SMI with the purpose of sense checking themes and findings. This involved one reviewer (HQ) having an informal conversation with each individual in which the initiation journey was discussed. Individuals were asked to comment on whether the review team’s interpretation of the data was clear and easy to understand.

### Search strategy

The bibliographic databases searched were ASSIA (ProQuest), CINAHL (EBSCO), Cochrane Central Register of Controlled Trials (Wiley), Health Technology Assessment Database (Wiley), MEDLINE (EBSCO), PsycINFO (ProQuest), Sociological Abstracts (ProQuest), SportDiscus (EBSCO), and Web of Science (Thomson Reuters, now Clarivate). Reviewers searched the author list and reference lists of all papers included in the review for other potentially eligible papers. No date limits were applied. Only papers published in the English language were included. The search strategy is identical to that published in the protocol [20], with the addition of two new terms “autobiographical” and “mental health” that the team recognized as necessary after initial searches.

### Search processes

Literature searches were undertaken in November 2017 by an experienced Information Scientist (DH). All results from the literature searches were exported to the bibliographic software, RefWorks (Ex Libris). This tool, instead of EndNote (Clarivate) as indicated in the protocol, was selected as a majority of the review team were familiar with this resource. RefWorks was also used to remove duplicate papers.

### Selecting primary studies

The selection process was divided equally among four reviewers (DH, EH, HC, and HQ) with a fifth reviewer (RC) available to advise on the overall approach. All papers were screened by one of the reviewers and 10% were independently double checked by a second reviewer. Eligibility criteria are described in detail by Quirk et al. [20]. In brief,

- **Population**—adults (≥18 years) living with SMI, defined as a primary diagnosis (as described in the studies) of schizophrenia, bipolar affective disorder, major depressive disorder, personality disorder, severe anxiety (including phobia and obsessive–compulsive disorder), schizophreniform disorder, or psychosis. If the population was described as those with SMI, but the specific condition was not reported, the paper was considered for review.
- **Intervention**—community-based group PA (inclusive of sport and exercise and any frequency, intensity or duration). The PA needed to take place in the community (i.e., those that take place outside of hospital, clinical, residential, or care settings), with a group being defined as a minimum of three people. If the intervention was multicomponent, PA needed to be a main component and the findings needed to be attributable to the PA.
- **Comparison**—if an intervention was described, no comparator condition was needed. Where a comparison was made, the comparator could be no activity or any other activity.
- **Outcomes**—qualitative data from the perspective of the participant living with SMI reporting the experience of initiating community-based group PA. Initiation was defined as the period in which people first start to engage in a PA. The initiation period could represent the first participation occasion or period of PA engagement, as long as findings could be attributable to the early phase of participation.
- **Setting**—community-based group setting.

### Outcome of study selection

**Figure 1.**
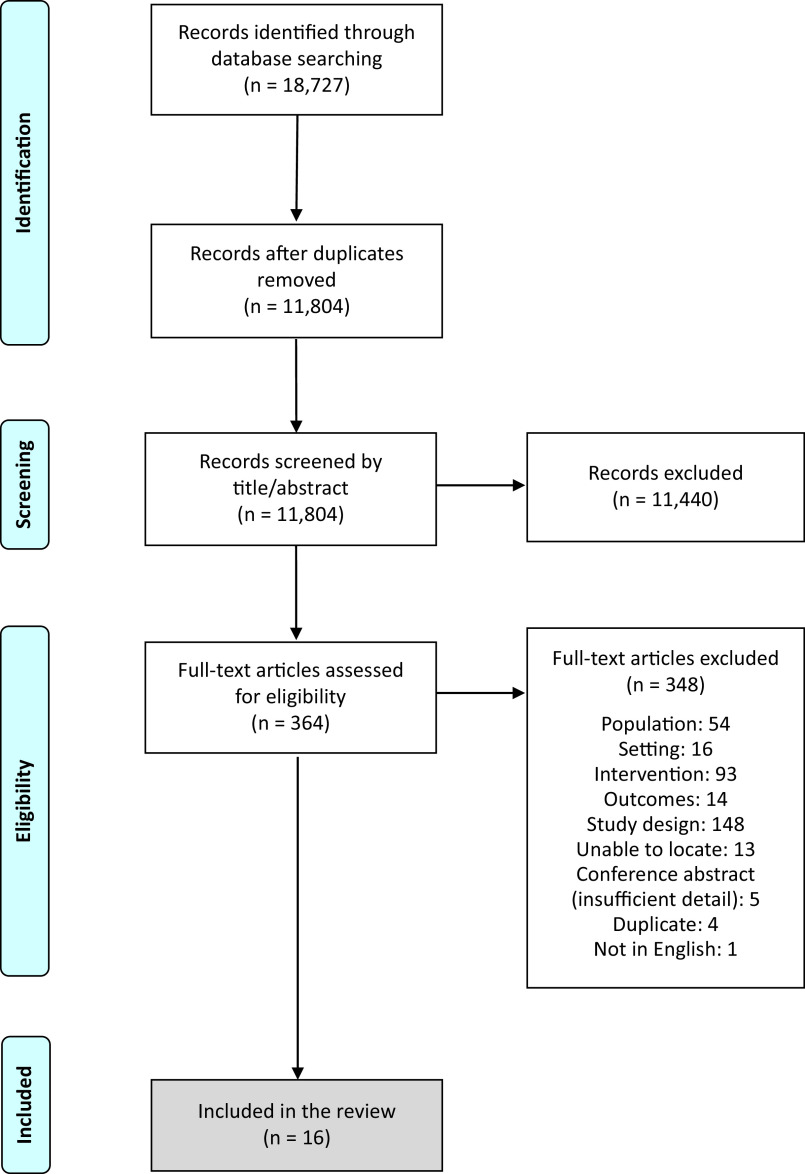
PRISMA flow diagram.

The database searches yielded 18,727 papers. After the removal of duplicates, there were 11,804 unique papers. All were screened using the title and abstract against the eligibility criteria. Following this process, 366 papers were retained for full-text screening. Sixteen papers met the eligibility criteria and were included in this review. A summary of the search and screening process is shown in the Preferred Reporting Items for Systematic Reviews and Meta-Analyses (PRISMA) flow diagram (Figure 1).

### Data extraction and quality appraisal

The data extraction method is outlined by Quirk et al. [20]. The extraction and quality appraisal document were piloted by the four members of the review team using two of the papers included in the review. Verbatim quotes from the findings and primary author statements were extracted and grouped into themes and subthemes, being careful to keep these distinct from the reviewers’ own comments or interpretations. Each reviewer’s data extraction tables were cross-checked by a second reviewer and any disagreement or discrepancies were resolved via discussion between the review team. The quality of each of the papers was assessed using the Critical Appraisal Skills Program (CASP) Qualitative Checklist [24]. Critical appraisals of all papers were cross-checked by a second reviewer and any discrepancies resolved via discussion among the review team.

### Translating second-order concepts

The outcomes extracted from primary studies were used to determine how studies were related (as per Noblit and Hare [22]) and identify the “key concepts”. The “key concepts” were themes that seemed particularly salient to the initiation of community-based group PA for people living with SMI. Each reviewer identified the key concepts in the studies they extracted and appraised. The key concepts were then copied into a “key themes and concepts” table, again preserving the meaning and source of the original text by copying quotes and study author comments verbatim and highlighting original participant quotes as first-order concepts, study author comments as second-order concepts, and reviewer interpretations as third-order concepts [25]. The “key themes and concepts” table helped the review team to identify patterns and relationships within and between studies. Translating second-order concepts was entirely grounded in the data. It was not guided by theory nor was the intention to substantiate or compare against previous theories or models of behavior change. For the same reason, our definition of initiation did not derive from any specific theory of behavior.

### Translating studies into one another

To generate third-order concepts, an Excel spreadsheet was created for each paper included in the study. The Excel spreadsheet outlined the first- and second-order concepts and a third column allowed the reviewer to add their own conceptualization of the theme/subtheme. Reviewers (HC, DH, EH, and HQ) followed step 5 of Noblit and Hare’s approach by exploring how the key themes and concepts related to each other. This was an iterative process determined by how much the studies agreed or disagreed with each other.

### Synthesizing translations

In meta-ethnography, the product of synthesis is the translation of study findings into one another to reach a new interpretation of the phenomenon being studied [23]. A series of review team meetings were held to synthesize the third-order concepts for each theme/subtheme. To ensure conceptualizations remained grounded in the original papers, cross-checks were continually made to the first- and second-order concepts. According to Noblit and Hare, studies can relate to one another in three ways: they may be directly comparable (reciprocal translations); they may stand in opposition to one another (refutational translations); or taken together they may represent a line of argument. A “line of argument” synthesis approach was used because the papers focused on different types of PA experiences that could usefully be brought together to construct overarching arguments about phenomena (stage 6; [22]). We did not identify any cases where concepts were strongly contested across papers (i.e., refutational translations [22]). Where findings from studies refuted other studies, these were noted.

### Expressing the synthesis

Key concepts identified across the papers, combined with a priority to retain the individual voice of people living with SMI, led to presenting this as a *“journey”* to visualize the complexity of the findings and identify potential phases of PA initiation. The phases of a person’s journey in the initiation of community-based PA were identified as: thinking about being active, planning and preparing for the activity, getting to the activity, and beginning the activity. In this case, the process of moving through the phases was cyclical, with people moving back and forth between phases as well as being static for extended periods of time. The service users viewed our interpretation as an accurate reflection of their own experience and likened our proposed journey to the “SMI recovery journey”.

### Confidence in cumulative evidence

Two reviewers applied the Confidence in the Evidence from Reviews of Qualitative Research (CERQual) tool to the review findings [26]. CERQual assesses confidence in evidence based on four components contributing to each review finding: the methodological limitations, the relevance, the coherence, and the adequacy of the data. The assessment of each component allows for a judgment of confidence; high, moderate, low, or very low. High confidence in a finding would suggest that it is highly likely that the review finding is a reasonable representation of the phenomenon of interest [26].

## Results

### Description of included studies

Sixteen studies (of 198 participants) were included for review. An overview of the characteristics of the studies is provided in Table 1. The studies were published between 2003 and 2017 and were conducted in the United Kingdom (*n* = 9), the United States (*n* = 3), Canada (*n* = 1), and Sweden (*n* = 1). Two did not report the location. A range of different PA types, interventions, and participants were represented. Nine studies included people living with a range of diagnoses [13,29,32,36,37,39–41]. Five studies included people living with schizophrenia or schizoaffective disorder [27,28,34,35,38] and four studies did not report the specific diagnosis, but instead reported a diagnosis of SMI [30,31,33].

**Table 1.** Study characteristics of included studies.

Reference and country	Study design and data collection	SMI diagnosis and length of diagnosis	Age		Gender			Demographics	Reported comorbidities/other health conditions
			Target	Mean (range)	Male (*n*)	Female (*n*)	NS (n)		
Bizub et al. [27], United States	NR (semi-structured interviews)	Within the schizophrenia spectrum	NR	NR (NR)	3	2	NA	Caucasian (*n* = 4), African–American (*n* = 1)	Personality disorders (*n* = 2), substance use disorder (*n* = 1)
Carless [28], United Kingdom	Interpretive ethnographic approach. In-depth interviews and participant observation	Schizophrenia/schizophrenic illness 4–8 years	NR	NR (NR)	4	0	NA	All were unable to live independently or to engage in paid employment.	NR
Carless and Douglas [29], United Kingdom	Case study method: “formal” semi-structured interviews, a focus group, “informal” interviews with clients, and participant observation	Severe and enduring mental health problems	NR	NR (NR)	9	0	NA	NR	NR
Carless and Douglas [30], United Kingdom	Narrative stories	No specific diagnosis reported	NR	NR (24–43)	11	0	NA	NR	NR
Carless and Douglas [31], Location NR	Participant observation with field notes and semi-structured interviews	No specific diagnosis reported	NR	NR (24–43)	11	0	NR	NR	NR
Carless and Douglas [32], United Kingdom	Narrative study	Serious and enduring mental illness such as schizophrenia or bipolar disorder (individual diagnoses not reported)	NR	NR (NR)	NR	3	NR	NR	NR
Carless and Sparkes [13], United Kingdom	Interpretive case study approach	SMI including schizophrenia 8–20 years	NR	NR (NR)	3	NR	NR	NR	NR
Crone [33], United Kingdom	Descriptive qualitative study	No specific diagnosis reported	NR	NR	2	2	NA	NR	NR
Evans [34], United Kingdom	NR	Schizophrenia	NR	NR (NR)	4	1	NA	All clients were dwelling in the community at the time of interview, rather than on secure wards	NR
Faulkner and Sparkes [35], United Kingdom	Ethnographic study	Schizophrenia with a long history (no further details reported)	NR	NR (“middle aged”)	2	1	NA	NR	NR
Graham et al. [36], Canada	Qualitative service evaluation	Participant demographics at 6 months: Schizophrenia spectrum disorders (*n* = 7); bipolar and related disorders (*n* = 5); depressive disorders (*n* = 6); anxiety disorders (*n* = 6); obsessive–compulsive disorders (*n* = 1); trauma and stress-related disorders (*n* = 2); substance and addictive disorders (*n* = 1). Participant demographics at 12 months: Schizophrenia spectrum disorders (*n* = 5); bipolar and related disorders (*n* = 2); depressive disorders (*n* = 4); anxiety disorders (*n* = 5); obsessive–compulsive disorders (*n* = 1); trauma and stress-related disorders (*n* = 1); substance and addictive disorders (*n* = 1); bipolar disorder (individual diagnoses not reported)	19+	50 (NR) (6 months)	4 (6 months)	17 (6 months)	NR	Living arrangements (n, % of sample): Rented accommodation (*n* = 20, 60.60%); own home (*n* = 7, 21.21%); own trailer (*n* = 3, 9.10%); live with family members (*n* = 6, 18.18%); live with caregiver (*n* = 1, 3.03%); living alone (*n* = 13, 39.39%); supported/assisted care arrangement (*n* = 3, 9.10%)	NR
Hodgson et al. [37], Location NR	Qualitative evaluation of an intervention delivered by the NHS and local authority	Severe and enduring mental illness under the care of the mental health service for 1–25 years	18–25	Male: 41.4 (NR); female: 43 (NR)	14	3	0	NR	NR
Hoffman et al. [38], United States	Qualitative evaluation of a locally delivered intervention	Diagnosis of schizophrenia or schizoaffective disorder as defined in the DSM-IV	18+	NR (NR)	6	8	NA	NR	No diagnosis of cognitive impairment and none other reported
Irving et al. [39], United Kingdom	Qualitative evaluation	Severe and enduring mental health problems (varying degrees of mental health problems).	NR	NR (NR)	NR	NR	NA	NR	NR
Wärdig et al. [40], Sweden	Qualitative exploratory study	Psychosis diagnosis (to include schizophrenia, schizoaffective disorder, bipolar, delusional disorder, unspecified psychosis) for 1–40 years	27–66	46 (27–66)	21	19	NA	NR	Established metabolic syndrome or at risk of developing the metabolic syndrome
Yarborough et al. [41], United States	Mixed-methods randomized control trial (qualitative arm formed part of the process evaluation)	Participants had diagnoses of schizophrenia or schizoaffective disorder (41%), bipolar disorder (20%), affective psychosis (37%), or PTSD (2%)	NR	48 (NR)	36%	64%	48	Ethnic or racial minority (*n* = 18, 21%) (oversampled minority group members at each time point); married or living with partner (*n* = 34, 41%); smoked all of last year (*n* = 20, 24%); income $0–$9,999 (*n* = 25, 31%); $10,000–$29,999 (*n* = 28, 35%); $30,000 or higher (*n* = 27, 34%); high school grad/GED or lower (*n* = 29, 35%); some college/technical (*n* = 37, 44%); college graduate or higher (*n* = 18, 21%); working (*n* = 22, 26%); disabled (*n* = 34, 41%); retired; unemployed; student; homemaker; temporarily laid off; or other (*n* = 28, 33%)	A BMI ≥ 27 to be eligible to participate

Abbreviations: BMI, body mass index; DSM-IV, 4th edition of the Diagnostic and Statistical Manual; NA, not applicable; NHS, National Health Service; NR, not reported; NS, (gender) not specified; PTSD, post-traumatic stress disorder; SMI, serious mental illness; UK, United Kingdom; USA, United States of America.

PA interventions varied across studies and included the following: walking group [33], walking and swimming [35], horse-riding [27], golf [29], swimming [34], low impact walking and yoga [36], and various activities including team sports [28,30–32,37–41]. The majority involved PA facilitated by an instructor or leader (*n* = 9). Five interventions were self-directed activities. In three studies, level of facilitation was unclear. See Table 2. for intervention characteristics. Descriptions of the interventions can be found in Supplement 1.

**Table 2.** Intervention characteristics of included studies.

Reference and country	Physical activity type	Self-initiated or intervention	Structured or unstructured	Facilitated or self-directed	Individual or group? (incl. size of group)	Intensity	Frequency	Duration or distance (of session/s)	Duration of intervention	Contact with mental health services or health professionals	Contact with which care workers
Bizub et al. [27], United States	Horse-riding	Intervention	Structured	Facilitated	Group (*n* = 5)	Low-moderate	Weekly	2 h (including the preparation and post-lesson processing group)	10 weeks	Clinical staff member (no further details)	“Volunteers” (no further details)
Carless [28], United Kingdom	Walking, running, gardening, gym-based exercise, soccer, badminton, swimming, and tennis	Both	Both	Both	Both: Activities in the day center were mainly group.	Various	Unclear	Unclear	Ongoing	Physiotherapists	NR
Carless and Douglas [29], United Kingdom	Sport (golf—made less competitive)	Intervention	Structured	Facilitated	Group (*n* = 9)	NR	weekly	NR	9 weeks	Support from mental health professionals in the form of phone calls	NR
Carless and Douglas [30], United Kingdom	Various	Unclear	Unclear	Unclear. Mainly facilitated? (details of golf group reported in Carless and Douglas 2004)	Unclear. At least two interventions were group (5-a-side football and golf)	Unclear	Unclear	Unclear	Unclear	NR	NR
Carless and Douglas [31], Location NR	Various exercise or sport activities, including golf, five-a-side football, badminton, tennis, swimming, walking groups, gardening, gym-based exercise, and running	Both	Both	Both	Both—mainly group	NR	NR	NR	NR	Day center, and mental health professional	A clinical psychologist, a senior physiotherapist, two care workers, two occupational managers, and an exercise leader.
Carless and Douglas [32], United Kingdom	Gym-based exercise, badminton, and tennis coaching sessions	Self-initiated	Structured for badminton and tennis (as coaching involved), not reported for gym.	Unclear	Badminton and tennis coaching/self-defense were group sessions.	NR	NR	NR	NR	NR	NR
Carless and Sparkes [13], United Kingdom	Gym-based exercise, football, badminton, walking, and swimming	Intervention	Both	Unclear	Individual engagement reported (some activities in groups, but details not clear).	NR	NR	NR	Engaging in exercise for at least 6 months at time of interview.	Physiotherapists and occupational therapists—mental health services not reported.	“Chaps would come round and take us out”. No other detail as to contact with care workers.
Crone [33], United Kingdom	Walking	Intervention	Structured	Facilitated	Group (unknown size)	NR	Monthly	NR	NR	NR	NR
Evans [34], United Kingdom	Aquatic leisure/swimming session	Both	Unstructured	Both	Group, clients and support workers.	NR	Weekly	1 hour	1 year	Support workers attended the swimming sessions with clients	Support workers
Faulkner and Sparkes [35], United Kingdom	Walking and swimming	Intervention	Structured	Facilitated	Group (*n* = 3)	Moderate	Twice per week	30 min	10 weeks	Unclear. Key workers were not present at exercise sessions	GF, the lead author, who was also a locum care worker at the hostel
Graham et al. [36], Canada	Walking, yoga, and low-impact fitness program	Intervention	Structured	Facilitated – by “peer leads”	Group (size not reported).	Exercise duration and difficulty were increased gradually and according to clients’ abilities	(a) Peer-led walking - Beginner: 2× weekly; Advanced: 2× weekly. (b) Yoga program—2× week. (c) Low-impact fitness program—1× week.	(a) Peer-led walking -average of 45 min. Advanced: 1 h 15 min. (b) Yoga program - NR. (c) Low-impact fitness program—60–90 min.	a) Peer-led walking—12 months. b) Yoga program—7 weeks. c) Low-impact fitness program - NR.	NR	NR
Hodgson et al. [37], Location NR	1) Men-only football; 2) “ACTIVE” program (15 sports and activity groups including basketball, tennis, walking, football, and badminton.	Intervention	Structured	Facilitated	Group PA (group size NR).	NR	NR	NR	Group 1 Men’s football - at least 3 months. Group 2 ACTIVE attendees - no requirement for length of involvement	NR	NR
Hoffman et al. [38], United States	Physical activity programs in the community	Unclear	NR	NR	Both	NR	NR	NR	NR	NR	NR
Irving et al. [39], United Kingdom	Physical activity/team games	Intervention	Structured	Facilitated	Group (average of *n* = 10 members)	NR	Weekly	About 1.5 h with a rest break.	3 years	NR	NR
Wärdig et al. [40], Sweden	Lifestyle intervention	Intervention	Unclear	Facilitated	Group (size NR)	NR	Weekly	NR	NR	NR	NR
Yarborough et al. [41], United States	Diet and exercise intervention	Intervention	Both	Both—but not clear	Both—eight groups/cohorts. Individual-based PA participation.	Unclear	Weekly (24 meetings)	Unclear	1 year	All sessions were coled by a mental health counselor and another interventionist familiar with nutrition interventions.	NR

Abbreviations: h, hour(s); min, minute(s); NR, not reported; PA, physical activity; SMI, serious mental illness; UK, United Kingdom; USA, United States of America.

### Quality of included studies

Using CASP, we judged that qualitative methods were appropriate in all studies and most studies had a clear statement of aims, an appropriate research design, data collection in a way that addressed the research issue, a clear statement of findings, and value in terms of practical application, and aiding our understanding of community-based PA for people living with SMI. Study quality varied in terms of having an appropriate recruitment strategy to support the aims, whether ethical issues were taken into consideration and if data analysis was sufficiently rigorous. Most studies did not adequately consider the relationship between researcher/s and participants. A summary of the methodological quality of studies is provided in Supplement 2.

### Synthesizing translations/line of argument

**Figure 2.**
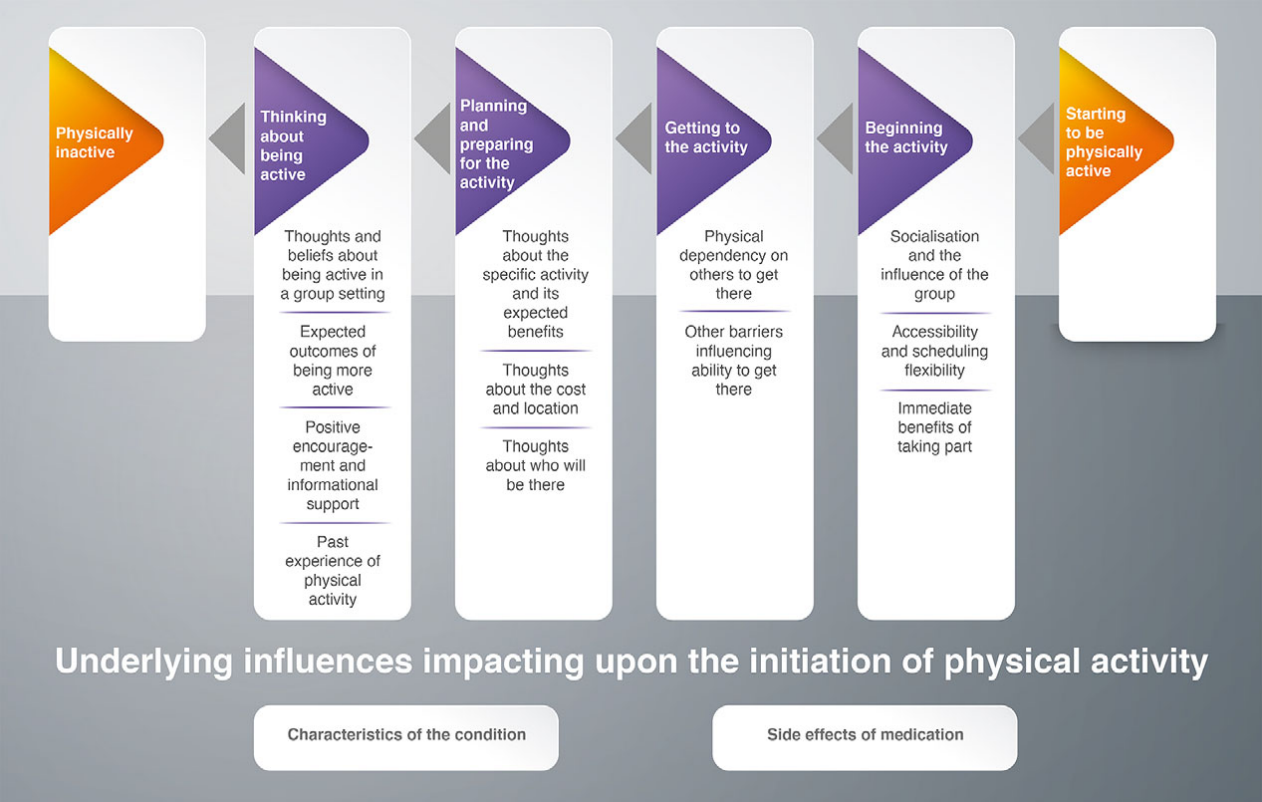
The journey of physical activity initiation for people living with SMI.

Table 3 shows a list of all the second-order constructs, using the original authors’ own words or a paraphrase to maintain the language used in each study [11,42]. When grouping second-order constructs into broader categories, we noticed a temporal sequence or “journey”. The “journey” captured a long sequence of phases which, although presented sequentially, is not linear in practice (Figure 2).

**Table 3.** First- and second-order construct table.

The journey used to frame and organize the second-order constructs	Summary definition (“translation”) of the phase of the journey	First-order constructs: illustrative quotations from participants in primary studies	Second-order constructs and summary definition (“translation”) of the second-order construct	Papers that include the second-order construct
Underlying influences impacting upon the initiation of physical activity	Underlying influences that can play a part in all stages of the physical activity initiation journey.	“It’s hard to make changes in your diet and follow the [exercise] routine…when you are at a point where you just do not care” (Yarborough [26], p. 6).	Characteristics of the condition: characteristics of the SMI and feeling “well enough” to engage in PA are underlying issues that influence all phases of the initiation journey. Characteristics of the condition include: poor body image, fatigue, low self-esteem, powerlessness, and the influence of fluctuations in the condition. Life is viewed through the lens of the condition, leaving little room for anything else.	Carless [28]; Carless and Douglas [32]; Crone [33]; Evans [34]; Irving et al. [39]; Wärdig et al. [40]; Yarborough et al. [41]
		“If you got your PC out and ran, like, 14 web searches and 8 lots of Photoshop and Word for Windows, it would gradually crank to a halt. And that’s exactly what going to the gym is like for me.” (Carless and Douglas [32], p. 168).		
		“… sometimes the actual drug treatments that you have that make you very tired, and makes it sometimes a struggle to actually get out of bed and do something” (Felix, 45 years). (Hodgson et al. [37], p. 26). “because being on my medication – I’ve put quite a bit of weight on and [physical activity] helps keep control of my weight as well because you get a bit self-conscious about that.” (Evans [34], p. 185).	Side-effects of medication: The side-effects of the antipsychotic medication make it difficult at all phases of the initiation journey because the individual does not feel “well enough” to participate. The negative side-effects of medication include tiredness, fatigue, and weight gain. In the initiation of PA, it is important to get the medication right before starting to be more active.	Carless [28]; Carless and Sparkes [13]; Evans [34]; Faulkner and Sparkes, [35]; Hodgson et al. [37]; Yarborough et al. [41]
Thinking about being active	The first phase of the journey in which people’s predisposing perceptions, beliefs, and values influence the decision about whether to engage in physical activity. These relate to general perceptions about physical activity and being more active, rather than beliefs about a specific type of physical activity.	“It’s like, putting myself in a position of vulnerability, having to meet lots of new things, people that aren’t necessarily predictable, I cannot always say who’s going to be there, or who’s not going to be there” (Carless and Douglas [32], p.167).	Thoughts and beliefs about being active in a group setting: Feelings of social isolation and vulnerability make it difficult to initiate PA in a community, group setting. Community settings are believed to be more unpredictable due to social anxiety and apprehension around strangers.	Carless and Douglas [32]; Evans [34]; Hodgson et al. [37]; Hoffman [38]
		“I have problems talking to strangers” (Evans [34], p. 182).		
		“It’s just that I’ve got an activity for the afternoon that I’m not sat watching TV something like that. I watch so much it just sort of draws me. I need to sort of break away from a day indoors and get out and do something … It’s something to get me out of bed, get out of bed that morning.” (Carless and Douglas [30], p. 583).	Expected outcomes of being more active: Thoughts about the outcomes of engaging in PA influence decisions about whether to take part in the activity. The expected outcomes need to be meaningful for the individual and outweigh the potential negative side-effects. Positive outcomes include a way of controlling symptoms, health improvement, a way of accessing clinical support/professional help, an opportunity to talk to others who share a similar experience, seeing friends or making friends, a worthwhile reason to get out of the house, and weight control. Negative outcomes include feeling vulnerable and insecure, stigma and embarrassment, problems with being coached/controlled by others, having to interact with others, having a panic attack, aches, pains, and sweating.	Carless [28]; Carless and Douglas [30]; Carless and Douglas [32]; Evans [34]; Faulkner and Sparkes [35]; Graham et al. [36]; Hodgson et al. [37]; Hoffman [38]; Irving et al. [39]; Wärdig et al. [40]; Yarborough et al. [41]
		“They [two physiotherapists] made a program for me and I started… I think they asked me what I wanted to do, but they just told me what was available and what I could fit in, like a school program.” (Carless and Douglas [31], p. 1186).	Positive encouragement and informational support: Social support is important when thinking about being active. Receiving positive encouragement to engage in PA and information about the benefits of the PA helps inform the decision about whether to take part. The source of the encouragement and information is important and could be health professionals, support workers, or family/friends.	Carless [28]; Carless and Douglas [31]; Carless and Sparkes [13]; Faulkner and Sparkes [35]; Hodgson et al. [37]; Hoffman [38]; Wärdig et al. [40]; Yarborough et al. [41]
		“I need someone to push me. I do not think I could ever do it on my own bat. I think I need somebody to give me that little push, to make sure that I do it, you know… It’s just having that person there to say, a member of staff or someone saying, go out and do yourself some good.” (Faulkner and Sparkes [35], p. 66).		
		“I was always playing football from the age of 16… we lived for football.” (Carless [28], p. 20).	Past experience of physical activity: Prior experience of PA plays an important role in the decision process about whether to take part. Positive experiences in past or before they were diagnosed with the SMI-facilitated initiation and helped the individual experience a sense of “normality”.	Carless [28]
Planning and preparing for the physical activity	Having thought about being more active, this phase includes thoughts about the specific activity and involves the individual considering the specific characteristics of the activity, including the expected benefits, the location, and who will be there. These thought processes all contribute to the individual developing a plan of action for the physical activity and developing a sense of feeling prepared to participate.	“I felt keen, you know, ‘cause I felt it was good time out and, you know, it’s not as if I’m playing a hectic sport, it’s pretty relaxed… It looked a very relaxed style sport – that’s the beauty of it.’” (Carless & Douglas [29], p. 34).	Thoughts about the specific activity and expected benefits: Individuals are influenced by specific characteristics of the activity being considered. In planning and preparing for the activity, individuals decide how desirable the activity sounds, including what the activity entails and the opportunities it provides.	Carless & Douglas [29]; Carless & Douglas [30]; Carless & Douglas [32]; Crone [33]; Evans [34]; Faulkner & Sparkes [35]; Hoffman [38]; Irving et al. [39]; Wärdig et al. [40]
		“The (sport center), it’s a beautiful track, it’s gorgeous, but…a person on disability cannot afford it. It’s a richman’s track.” (Graham et al. [36], p. 844).	Thoughts about the cost and location: In planning and preparing for the activity, individuals consider the direct costs of participation in the activity and whether it is affordable to them. Consideration is also given to the location of the activity and how suitable or appropriate it is believed to be for the individual or “people like us”.	Carless & Douglas, [29]; Carless & Douglas [31]; Crone [33]; Graham et al. [36]; Hodgson et al. [37]; Wärdig et al. [40]
		“If it wasn’t for Sarah and Catherine [two physiotherapists] I do not think I’d have got back into it. Well, I would have got back into it, but not so soon… I think it was important for them to be there first of all. It gave me a bit of confidence. Because I was so unwell, I would not have had no confidence, thinking I was gonna have a panic attack, stuff like that… somebody there I could chat to and take my mind off it and stuff.” (Carless and Douglas [31], p. 1189).	Thoughts about who will be there: In planning and preparing for the activity, individuals consider the other people who will be there and the expectations of how the individual will feel in the company of others. Thoughts about who will be there relate to the other PA participants and the staff members/supervisors involved in the delivery of the activity or working at the facilities.	Carless and Douglas [31]; Carless and Douglas [32]; Evans [34]; Faulkner and Sparkes [35]; Hodgson et al. [37]; Irving et al. [39]; Wärdig et al. [40]
Getting to the activity	Having considered the details of the specific activity, this phase involves the individual actually getting to the activity. It is expected that the individual has a plan for participation and has moved into the phase of actually getting to the activity.	“I do not actually come on the bus, Sally picks up Maureen first and then she picks me up after, and then we come in the car, so really, I do not know how we’d get here otherwise because I’m not able to get on a bus on my own” (Hodgson et al. [37], p. 26).	Physical dependency on others to get there: Having tangible support in place physically enables individuals to get to the activity and facilitate the initiation of PA. It can involve reminders (e.g., telephone calls), help with transport, transport costs, or accompaniment to the activity. Often the support comes from health professionals or support workers but can also come from friends/family.	Carless 2007 [28]; Carless and Douglas [29]; Carless and Douglas [31]; Crone [33]; Hodgson et al. [37]; Hoffman [38]; Irving et al. [39]
		“I have a bit of difficulty with motivation of going on my own and I’ve not really been able to manage going on my own so I appreciate going [swimming] with them [the support workers]” (Evans [34], p. 182).		
		“And then I have my daughter as well. I cannot go out when she comes home and I cannot do anything when she’s at home. We’re stuck in the house, like a prison.” (Wärdig et al. [40], p. 606).	Other barriers influencing ability to get there: Other barriers can include personal responsibilities or other commitments such as family life including childcare also make getting to the activity difficult. Other fears and concerns may make the journey to the activity difficult.	Carless and Douglas [29]; Crone [33]; Hoffman [38]; Wärdig et al. [40]
Beginning the activity	This phase refers to the phase in the individual’s journey when the initial uptake occurs (i.e., they take part for the first time). It is expected that by this phase, the individual has thought about being more active, planned and prepared for a specific activity, got to the activity, and has arrived at a place (physically and emotionally) of feeling ready to actually participate.	“Well you are meeting other people that are sharing a common thing aren’t you really? Common exercises, sharing that experience. That’s what I reckon anyway. So it’s good on that side of it…all doing the same thing, got the same experience and got something to talk about.” (Carless and Douglas [30], p. 587).	Socialization and the influence of the group: When beginning to be active in a group setting, individuals engage with the other people present. The initial experience is often affected by the other participants or the health professionals, support workers, or supervisors. Sharing the experience with others deemed similar to oneself and elicits mutual understanding and creates a nonjudgmental atmosphere. However, not all individuals will benefit from the socialization opportunities provided through group-based PA.	Bizub et al. [27]; Carless and Douglas [30]; Carless and Douglas [32]; Crone [33]; Faulkner and Sparkes [35]; Irving et al. [39]; Wärdig et al. [40]; Yarborough et al. [41]
		“When I self-harm, I feel less judged on my bruises/marks at the badminton group than I do with other people. Like when I go to my volunteering at the charity shop, I wear long sleeves to cover my arms, whereas at badminton I feel comfortable wearing a t-shirt. Another thing is, if we talked too much about our illness/problems to so-called “normal people” they might think we are a bit self-obsessed, whereas in the badminton group because we all have similar problems, it is good to share it with each other. It’s good because nothing is expected of you. You take it at your own pace. If you are having a bad day and just feel like watching, that is OK.” (Carless and Douglas [32], p. 168).		
		“I love the fact that there’s different groups for different people. So it’s tailored for everybody’s needs.” (Graham et al. [36], p. 844).	Accessibility and scheduling flexibility: When beginning to be active, the extent to which the activity can be tailored to different levels of ability and ages is important. Individuals value having a sense of control over the level at which they participate. Program schedules that are flexible to other commitments or relapses the individual may might experience are beneficial.	Carless and Douglas [32]; Crone [33]; Evans [34]; Graham et al. [36]
		“I would be meeting other people and it would be very relaxing and you could just get into the pool and do what you wanted and there would be nobody hassling you or following you. You could be just relaxed and be with your own thoughts.” (Evans [34], p. 186).	Immediate benefits of taking part: The immediate feelings or perceived benefits experienced as a result of engagement in the physical activity are important for ongoing participation. These include mood enhancement, sense of freedom, relaxation, sense of achievement, and self-appreciation. However, emotions such as apprehension or social anxiety can also occur in group-based PA.	Bizub et al. [27]; Carless [28]; Carless and Sparkes [13]; Crone [33]; Evans [34]; Faulkner and Sparkes [35]; Wärdig et al. [40]
		“a bit proud of myself. I was actually doing something that was worthwhile and slightly constructive.” (Faulkner and Sparkes [35], p. 63).

### The journey of PA initiation for people living with SMI

Table 3 gives a narrative “translation” of each second-order construct.

## Underlying Influences Impacting upon the Initiation of PA

Two subthemes were identified representing underlying influences that play a part across the initiation journey. Characteristics of SMI that affected people’s ability to initiate PA were low self-esteem, feelings of failure, and/or powerlessness—especially after a failed attempt at initiating PA [34,40,41]. For some people dealing with SMI was all-consuming and left little space in their life for anything else [32,40].

Another challenge was side-effects of medication that caused people to lack motivation, feel drowsy and sluggish, or too ill to participate in PA [13,34,35,37,41]. Getting the medication right to feel “well enough” to partake in PA was important to reduce any setbacks in PA initiation [13,28]. Weight gain or being overweight was considered a side-effect of medication and was spoken about in relation to feelings of poor body image and insecurity [32,34,35].

## Thinking About Being Active

In the first phase of the journey, predisposing perceptions, beliefs, and values influence the decision to engage in PA. These relate to perceptions about PA in general, rather than beliefs about a specific type of PA, which come later in the journey. Four subthemes were identified within this phase of the journey: *thoughts and beliefs about being active in a group setting*, *expected outcomes of being more active*, *positive encouragement and informational support*, and *past experience of PA.* People living with SMI talked about feeling vulnerable in unpredictable group settings due to social anxiety, embarrassment, and apprehension around strangers [32,34,36–38,40].

While the perceived benefits of PA varied across studies (e.g., a worthwhile reason to get out of the house [or other residential setting] [13,28,34,39] and weight control [28,35,38,40,41]), expected outcomes needed to be meaningful and outweigh the perceived negative effects for individuals to move to the next step in the journey. Positive encouragement from trusted sources was important when people were thinking about increasing PA [31,35]. This included initial awareness-raising via information-giving from health professionals (e.g., about the benefits of PA) and verbal persuasion [13,28,31,38]. On the whole, people living with SMI understood the benefits of PA [38,41], so information about what was available in their local community and encouraging people to engage with what was available was most beneficial. Finally, having a previous positive experience of PA was also beneficial when thinking about being active, providing a sense of “normality” for those who had been active prior to their SMI diagnosis [28].

## Planning and Preparing for the PA

Having thought about being more active, the planning and preparing phase of the journey involves developing an action plan and preparing for the activity. Three themes were identified within this phase: *thoughts about the specific activity and its expected benefits*, *thoughts about cost and location*, and *thoughts about who will be there.* In thinking about activities, people living with SMI considered how desirable the specific activity seemed compared to alternative options [29,33–35]. Often, the expected benefits of community-based PA related to having something to do and somewhere to go, rather than specific to the activity itself; doing something is better than doing nothing [30,33–35,39].

People living with SMI often felt unable to participate due to the activity being financially or physically inaccessible [31,37,40]. Activities had to be affordable to be perceived as sustainable [29,36]. People living with SMI benefited from feeling familiar with the location and easily being able to travel the distance to get there, as well as feeling they belong at the location [36].

Thinking about socializing with others, including staff delivering and facilitating the session, was appealing to some [32,35,37], but others felt apprehensive about having to have conversations with other people [34,39,40]. The presence of trusted and known health professionals or members of the mental health support team was valued and believed to instill confidence among people living with SMI initiating a new PA [31–33,35].

## Getting to the Activity

The next phase involves putting plans into action. Two themes were identified within this phase of the journey: *physical dependency on others to get there* and *other barriers influencing ability to get there.* People living with SMI often depended on intensive support from other people to get out of the house (or other residential setting) and to the activity (e.g., reminders, transportation, travel expenses [38], and accompaniment [28,29,31,33,39]). Personal responsibilities or other commitments such as family life and childcare also made getting to the activity difficult [33,38,40].

## Beginning the Activity

This phase refers to taking part for the first time. Three themes were identified: *socialization and the influence of the group, accessibility and scheduling,* and *immediate benefits of taking part.* The first experience was influenced by the other people present. A supportive atmosphere and feeling safe in the company of others fostered connectedness and mutual understanding [30,32–35,40,41]. Welcoming and supportive staff instilled confidence [27,32,33,35,39]. People living with SMI benefited from knowing what to expect before arriving and from knowing that the schedule was adaptable to their needs (e.g., symptoms, health relapses, and ability). They also valued autonomy to decide their level of participation [32–34,36]. The immediate perceived benefits of taking part such as enjoyment or mood enhancement, relaxation, and sense of achievement were important contributors to the successful initiation of PA [13,28,33–35,40].

### Confidence in the cumulative evidence

The CERQual assessment of our level of confidence in the findings indicated that more and/or better quality primary research is needed in this area. On the basis of our CERQual assessment of the review findings, we have moderate confidence in 11 findings and low confidence in three findings (see Table 4. for the CERQual assessment).

**Table 4.** CERQual summary of qualitative findings table.

Review finding	Studies contributing to the review finding	CERQual assessment of confidence in the evidence	Explanation of CERQual assessment
Characteristics of the condition	Carless [28]; Carless and Douglas [32]; Crone [33]; Evans [34]; Irving et al. [39]; Wärdig et al. [40]; Yarborough et al. [41]	Moderate confidence	Seven studies contribute to this finding. Moderate methodological limitations, moderate concerns about adequacy with thin data in six studies, and moderate concerns about relevance due to some studies not being all community-based and/or all group-based. Minor concerns about coherence.
Side-effects of medication	Carless [28]; Carless and Sparkes [13]; Evans [34]; Faulkner and Sparkes [35]; Hodgson et al. [37]; Yarborough et al. [41]	Moderate confidence	Six studies contribute to this finding. Moderate methodological limitations and moderate concerns about adequacy due to fairly thin data. Minor concerns about coherence and relevance.
Thoughts and beliefs about being active in a group setting	Carless and Douglas [32]; Evans [34]; Hodgson et al. [37]; Hoffman [38]	Low confidence	Four studies contribute to this finding. Serious methodological limitations and moderate concerns about adequacy (due to lack of rich data) and relevance. Minor methodological limitations.
Expected outcomes of being more active	Carless [28]; Carless and Douglas [30]; Carless and Douglas [32]; Evans [34]; Faulkner and Sparkes [35]; Graham et al. [36]; Hodgson et al. [37]; Hoffman [38]; Irving et al. [39]; Wärdig et al. [40]; Yarborough et al. [41]	Moderate confidence	Eleven studies contribute to this finding. Moderate methodological concerns, moderate concerns about adequacy due to fairly thin data, and moderate concerns about relevance due to some studies not being all community-based and/or all group-based. Minor concerns about coherence.
Positive encouragement and informational support	Carless [28]; Carless and Douglas [31]; Carless and Sparkes [13]; Faulkner and Sparkes [35]; Hodgson et al. [37]; Hoffman [38]; Wärdig et al. [40]; Yarborough et al. [41]	Moderate confidence	Nine studies contribute to this finding. Moderate concerns about adequacy and relevance. Minor methodological limitations and minor concerns about coherence.
Past experience of physical activity	Carless [28]	Low confidence	One study contributes to this finding. Moderate methodological limitations and moderate concerns about adequacy and relevance. Minor concerns about coherence.
Thoughts about the specific activity and expected benefits	Carless and Douglas [29]; Carless and Douglas [30]; Carless and Douglas [32]; Crone [33]; Evans [34]; Faulkner and Sparkes [35]; Hoffman [38]; Irving et al. [39]; Wärdig et al. [40]	Low confidence	Nine studies contribute to this finding. Serious methodological limitations and moderate concerns about adequacy and relevance. Minor concerns about coherence.
Thoughts about the cost and location	Carless and Douglas [29]; Carless and Douglas [31]; Crone [33]; Graham et al. [36]; Hodgson et al. [37]; Wärdig et al. [40]	Moderate confidence	Six studies contribute to this finding. Moderate methodological limitations, moderate concerns about adequacy due to fairly thin data in most studies (although it seems well-established), and moderate concerns about relevance. No/very minor concerns about coherence.
Thoughts about who will be there	Carless and Douglas [31]; Carless and Douglas [32]; Evans [34]; Faulkner and Sparkes [35]; Hodgson et al. [37]; Irving et al. [39]; Wärdig et al. [40]	Moderate confidence	Seven studies contribute to this finding. Moderate methodological limitations and moderate concerns about relevance. Minor concerns about coherence and adequacy.
Physical dependency on others to get there	Carless [28]; Carless and Douglas [29]; Carless and Douglas [31]; Crone [33]; Hodgson et al. [37]; Hoffman [38]; Irving et al. [39]	Moderate confidence	Seven studies contribute to this finding. Moderate methodological limitations and moderate concerns about relevance. Minor concerns about coherence and adequacy.
Other barriers influencing ability to get there	Carless and Douglas [29]; Crone [33]; Hoffman [38]; Wärdig et al. [40]	Moderate confidence	Four studies contribute to this finding. Moderate methodological limitations and moderate concerns about relevance. Minor concerns about coherence and adequacy.
Socialization and the influence of the group	Bizub et al. [27]; Carless and Douglas [30]; Carless and Douglas [32]; Crone [33]; Faulkner and Sparkes [35]; Irving et al. [39]; Wärdig et al. [40]; Yarborough et al. [41]	Moderate confidence	Eight studies contribute to this finding. Serious methodological limitations. Moderate concerns about adequacy, as some studies had thin data, and relevance, as not all settings may have been community-based and not all activities may have been group-based, although this seems less important for this finding. Minor concerns about coherence.
Accessibility and scheduling flexibility	Carless and Douglas [32]; Crone [33]; Evans [34]; Graham et al. [36]	Moderate confidence	Four studies contribute to this finding. Moderate methodological limitations, moderate concerns about adequacy due to mainly thin data, and moderate concerns about relevance. Very minor concerns about coherence.
Immediate benefits of taking part	Bizub et al. [27]; Carless [28]; Carless and Sparkes [13]; Crone [33]; Evans [34]; Faulkner and Sparkes [35]; Wärdig et al. [40]	Moderate confidence	Seven studies contribute to this finding. Moderate methodological limitations, and moderate concerns about relevance, as not all settings may have been community-based, not all activities may have been group-based, and population was not clearly specified in one study. Minor concerns about coherence and adequacy.

## Discussion

The current review aimed to explore how adults diagnosed with SMI experience the initiation of community-based group PA and key features of social support within these contexts. Findings illustrate that initiation of PA in community group-based contexts is not a simple step from intention to participation. The journey is not always linear, but a slow process with challenges or setbacks at every phase. Similar to Soundy et al. [11], our findings demonstrate the challenges, complex processes, and facilitators that exist before PA even begins for people with SMI. These are associated with thinking about being active, planning and preparing for PA, getting to the activity, and beginning the activity. The journey we present enhances the existing evidence base by providing in-depth exploration of the complexities of the initiation process specific to community-based group PA.

We identified some of the benefits and challenges that characterize group PA in the community setting for people living with SMI. Beneficial qualities include having a reason to get out of the house (or other residential setting), socializing, and feeling connected with others. Challenges involve feelings of vulnerability and social anxiety, dependency on others to provide or pay for transport, and the perceived appropriateness of the activity setting. Furthermore, the cost of some activities (e.g., golf) may preclude participation compared to lower cost activities. People also benefited from knowing what to expect from the activity and facilities prior to attending and we identified a need for a supportive, safe, and nonjudgmental atmosphere. Flexibility in scheduling of PA was also important, as reflected in the UK practitioner guidelines for people working in mental health services [43].

The current review highlighted the importance of taking an individualized approach to PA promotion in people with SMI. This could be likened to a person-centered care approach [44, 45] and in that it takes into consideration an individuals’ values, self-identify, family situation, social circumstances, past experiences, beliefs, and preferences as well as medication, motivation, available support, and cost of the activity (including transport and other associated costs). Expectations about the outcome of the activity can impact whether or not, how, and when people living with SMI initiate PA. Previous findings from quantitative studies in this population have identified health-related outcomes as important motivators for PA engagement [19]. Our findings support health-related outcomes such as weight control as important motivators, but also suggest that the desired outcome of the PA might be more about having somewhere to go and something to do.

The intensive nature of the social support required during the initiation of community-based group PA for people living with SMI is a key outcome from this study. The need for instrumental and informational support has been demonstrated previously [14], as has emotional support [46]. We identified that sources of support need to come from someone who is valued and respected by the person living with SMI and someone who knows them well and is well known to them, which supports previous findings [46]. Consistent with previous research, this could be provided by mental health professionals such as physiotherapists and occupational therapists [47]. While some people with SMI are comfortable in accepting support from professionals, others do not want this [48] and prefer support from trusted caregivers, friends, and family [49].

Social support from others living with SMI was also found to be important as it provided a supportive and “safe” atmosphere. This was particularly important for those with low self-image and confidence, providing a sense of togetherness and shared identity of doing something “normal” without feeling judged or stigmatized. This supports previous findings about the importance of shared identities by people attending group-based PAs [11,33,46]. Providing people with SMI with a safe environment within which to make choices and decisions about their participation could translate into benefits in different areas of life [11,14,46]. Carless and Douglas [50] likened this to having a door opened that is usually shut for people living with SMI.

## Implications for Future Research

This review has demonstrated the importance of intensive social support in the initiation of community-based group PA. It is not clear, however, how long this intensive support and close interaction is needed to facilitate successful initiation of the activity. Further research should explore whether the support needs to be consistent for the duration of participation and the implications of a break or change in the level of support provided. Carless [28] suggests that progress from initiation to maintenance of PA among people living with SMI can take years rather than weeks or months (as per a more traditional definition of initiation [51]). Using qualitative research to really understand the unique experience of the journey preceding each PA session is needed to help ensure the sustainability of programs and related outcomes. Further research exploring the continuation of PA, to establish the extent to which the factors involved are similar to those for initiation, appears warranted.

Many of the findings included in our review were derived from bespoke interventions that involved services that would not normally be available for people living with SMI in the community (e.g., golf lessons and exclusive access to a swimming pool). A recent position statement for PA as treatment for SMI by Stubbs and colleagues [10] has called for “*replicable and scalable methods for delivering PA interventions to people living with SMI, in a format which is accessible, engaging, and effective for large numbers of patients*” (p. 140). It is currently unclear whether PA is a cost-effective treatment option for people living with SMI and more work is needed to establish whether the financial implications are offset by the benefits [10]. This raises important questions about the extent to which the findings included in this review can be generalized to the broader community of people living with SMI and suggests that further research is needed to explore the experience of community-based PA initiation in everyday life.

## Strengths and Limitations

This study benefits from a rigorous application of method, conducted according to the Noblit and Hare meta-ethnography approach. The manuscript also adheres to the reporting guidelines by France et al. [23] and guidance such as [52]. Most notably, service users felt reassured that the way the review captured the initiation of PA (e.g., a slow complex process rife with problems, barriers, and setbacks that is heavily reliant on the support of others) was a “real life” representation [53]. This is a particular strength of this study.

The findings should be considered in light of some methodological limitations. Our inclusion of manuscripts written in English language may have missed important research reported in other languages. Similarly, all studies were conducted in western, developed countries with no studies conducted in developing countries. The demographic characteristics (e.g., ethnicity) of participants were reported poorly, in part due to the need to protect confidentiality of participants. This means there has been no exploration of differences in the experience of initiation of PA by demographic variables, which is worthy of further research.

This review included papers with combined results and discussion sections, which makes meta-ethnographic analysis difficult due to a lack of clarity about what is a finding (first-order concept) and what is the primary authors’ interpretation (second-order concept), limiting the ability to make third-order interpretations. Despite this, as far as possible, we distinguished between first and second-order constructs in the data extraction phase and can demonstrate that the papers with mixed results and discussion sections have added value (e.g., Carless [28]; Crone [33]). Crone [33] argued that the integration of findings and discussion allows the development of links between analytic categories and wider issues of theory.

## Recommendations for Practice

We make the following recommendations for practice based on the findings from the current review. Extra support and resource allocation may be necessary in the “getting to the activity” phase of the journey, while ensuring that there is no disadvantage toward individuals in terms of cost of participating in the activity or ongoing participation. Financial cost has been a strong theme in previous literature [10,11].

Promotion of PA would benefit from taking an individualized approach that is “pitched” appropriately and takes into consideration the person as a whole and the social support networks they have around them.

Providers should be sure that people living with SMI have their preferred level of support throughout the journey, both physically and emotionally, and that facilities are welcoming, nonstigmatizing, and make users feel safe and secure. The people providing support should ideally be known to the individual with SMI, preferably valued, and respected by them.

Flexibility regarding attendance, scheduling, and rate of progression appears central to PA initiation. Priority should also be given to promoting enjoyment, choice, autonomy, and decision making in the very early phases of the PA initiation journey. To allow for this flexibility, traditional measures of success for PA programs may need to be changed. For example, focusing on attendance rates may not be an ideal indicator of success in a program with flexible scheduling and relaxed attendance expectations.

## Conclusion

The outcomes and subsequent “journey” presented in this review provide an authentic narrative of the lived experience of the initiation of community-based group PA for people living with SMI. It illustrates that initiation of PA is a complex process rife with challenges. It is not a simple step from intention to participation. The process of initiating PA among people with SMI and the facilitating factors demonstrated here should be fully understood before issues of ongoing participation can be addressed. The literature has demonstrated that alongside the complexity of the journey and the need to take an individualized approach to PA initiation, encouraging people living with SMI into community-based group PA requires high resource, in terms of time, transport, cost, and professional supervision. In particular, intensive social support from a trusted source (health professionals, providers, friends, and family) has an important role in getting people to the activity both physically and emotionally. This review provides recommendations for practice that could inform future PA programs and optimize the uptake of community-based group PA among people living with SMI.

## Supplementary material

For supplementary material accompanying this paper visit https://doi.org/10.1192/j.eurpsy.2020.93. click here to view supplementary material
