# Supplementary material for: Understanding the experience of initiating community-based group physical activity by people with serious mental illness: A systematic review using a meta-ethnographic approach
Source: Eur Psychiatry. 2020 Oct 22;63(1):e95. doi: 10.1192/j.eurpsy.2020.93 (PMC7681136; doi:10.1192/j.eurpsy.2020.93)
Supplement: Supplementary file 1 [file S0924933820000930sup.zip › S0924933820000930sup01.docx]

**Supplement 1: Description of interventions in included studies**

| **Reference & country** | **Physical activity type** |
| --- | --- |
| Bizub et al (2003) USA [37] | The programme was divided into three parts: 1) bonding activities with the horse; 2) mounted activity; and 3) post-riding processing group. To begin, the five individuals would ride together to High Hopes. Upon arrival, participants prepared their horses by grooming them with the volunteers, then engaged in mounted activity that included both lessons in basic horsemanship (learning commands such as “whoa” and “walk on,” steering a horse and posting to the trot) and trail rides. Volunteers stayed with the riders during the lesson and there typically were two volunteers per rider: one holding the horse by the lead rope and the other walking alongside the horse and rider. Finally, after dismounting and returning the horses to their paddocks, riders engaged in a post-lesson processing group that utilised creative exercises to facilitate sharing. |
| Carless (2007) UK [36] | Participants had begun participating in exercise under the guidance of physiotherapists at a mental health rehabilitation day centre, and the exercise sessions were an integral component of each participant’s weekly schedule. Subsequent to this, some participants did self-directed forms of exercise. |
| Carless & Douglas (2004) UK [33] | Structured nine-week golf intervention designed to support task competence and enjoyment. Coaching input was provided throughout the programme, in line with our ethos of facilitating a positive golf experience for the group members. Group members were provided with free minibus transport, free entry to the golf facilities, free equipment, and free tea or coffee and biscuits after the session. Additionally, some were telephoned before each session to remind them, and others were collected from their homes. |
| Carless & Douglas (2008a) UK [40] | Included: A five-a-side football group, a golf activity group. Included anything organised that gave participants a reason to go out. Structure of golf group reported in Carless & Douglas (2004). |
| Carless & Douglas (2008b) Location NR [39] | The golf activity was described in Carless & Douglas (2004). Mental health professionals were closely involved in each activity session in an organisational capacity and followed professional guidelines for safe working practice. |
| Carless & Douglas (2012) UK [32] | Badminton coaching group, tennis coaching group, self-defence group, gym. |
| Carless & Sparkes (2008) UK [13] | Gym based exercise, football, badminton, walking, swimming. Unclear if all these activities were group-based. Badminton tournament reported to be organised by the physiotherapists. |
| Crone (2007) UK [41] | Group walks taking place within the county of Somerset, UK, monthly. The group travelled to the location of the walk, which was pre-planned and arranged by the project coordinator. Locations for the walks included both picturesque and educational settings, for example, Areas of Outstanding Natural Beauty and land owned or managed by organisations such as Somerset Wildlife Trust and the Royal Society for the Protection of Birds (RSPB). Walks often included educational talks from guides about a range of interests including wildlife, the herbal usage of plants, and fauna and flora. The walks took place in a variety of locations, ranging from trails in the woods, lakes, and on coastlines. |
| Evans (2017) UK [35] | Ongoing aquatic leisure sessions were tailored to cater for participants diagnosed with severe and enduring mental ill health, together with their support workers. These sessions had taken place weekly at one pool for a one-hour period for a period of one year prior to interviews, in a city in the northeast of England, and which were attended by clients on a voluntary basis for as long as the scheme was funded; it had been running for over a year at the time of interviews. During sessions, participants, referred to as ‘clients’ had sole use of the pool. They were also supported by a lifeguard and a swimming instructor during their time in the pool, in addition to their support workers. The instructor was present to provide advice if clients wished, but sessions had remained otherwise unstructured and could be used as clients wished. |
| Faulkner & Sparkes (1999) UK [38] | Ten-week exercise programme, consisting of 30 minutes of continuous moderate activity, twice per week. Participants chose walking in nearby parks and swimming at the local leisure centre. Scheduled sessions were led by the lead author (GF) in the late afternoon. |
| Graham et al (2017) Canada [31] | a) Peer-led walking programme held at two community sports centres– beginner and advance groups – indoors or outdoors dependent on weather and preference. b) Yoga programme at local yoga studio informed by needs of mental health service users and led by qualified instructor. Peer leads helped organise the class scheduling and the practice setting. c) Low impact fitness programme – informed by need of mental health service users and led by peer leads. |
| Hodgson et al (2011) Location NR [30] | ‘ACTIVE’ programme attendees – ACTIVE is an established citywide, community-based sport and physical activity programme. It runs in partnership between the Local Authority and the Physiotherapy and Exercise department within a mental health trust in the NHS. It incorporates 15 sports and activity groups throughout the week including basketball, tennis, walking, three football and four badminton groups. Each group is run by a qualified sports coach and supported by a mental health professional. The ‘ACTIVE’ sessions are subsidised by an external grant. |
| Hoffman et al (2014) USA [34] | Physical activities delivered at the community-based centre offering residential programming for individuals living with mental illness |
| Irving et al (2003) UK [29] | The group met in a hired hall on a weekly basis from 1999 to 2002, with an average of ten members and two staff. Most weeks, the group chose team games as the dominant activity and these lasted for about an hour and a half with a rest break. |
| Wärdig et al (2013) Sweden [28] | The intervention followed the compendium “Solutions for Wellness,” which focuses on theoretical education regarding healthy eating and other lifestyle issues, such as smoking, alcohol habits and physical activities. Physical exercise was based on the individual’s own abilities and performed in groups under the guidance of health coordinators trained in motivational interviewing. |
| Yarborough et al (2016) USA [27] | The STRIDE intervention was an adaptation of the PREMIER comprehensive lifestyle intervention that promoted the DASH dietary eating pattern and was tailored for individuals taking antipsychotic medications. The intervention consisted of 24 weekly meetings that targeted readiness to change; included interactive, participant-centred delivery of lifestyle education information along with a 20-minute walk; encouraged skills practice, self-monitoring and feedback; and facilitated group interactions and support. Intervention participants could consult with interventionists by telephone as needed. Participants were encouraged to keep detailed weekly logs of calories consumed, exercise, and sleep Interventionists reviewed logs with participants weekly and gave feedback. Six monthly group maintenance sessions followed the weekly meetings. |

NR, not reported; PA, physical activity; SMI, serious mental illness; UK, United Kingdom; USA, United States of America
