# Supplementary material for: Understanding the experience of initiating community-based group physical activity by people with serious mental illness: A systematic review using a meta-ethnographic approach
Source: Eur Psychiatry. 2020 Oct 22;63(1):e95. doi: 10.1192/j.eurpsy.2020.93 (PMC7681136; doi:10.1192/j.eurpsy.2020.93)
Supplement: Supplementary file 1 [file S0924933820000930sup.zip › S0924933820000930sup02.docx]

**Supplement 2: Quality assessment of included studies using CASP Qualitative Checklist**

| **Study** | **Clear statement of aims?** | **Qualitative methods appropriate?** | **Research design appropriate to address aims?** | **Recruitment strategy appropriate to aims?** | **Data collected in a way that addressed research issue?** | **Relationship between researcher and participants adequately considered?** | **Ethical issues taken into consideration?** | **Data analysis sufficiently rigorous?** | **Clear statement of findings?** | **Does the research have practical application?** | **Does it add to our understanding?** |
| --- | --- | --- | --- | --- | --- | --- | --- | --- | --- | --- | --- |
| Bizub et al (2003) USA [37] |  |  |  |  |  |  |  |  |  |  |  |
| Carless (2007) UK [36] |  |  |  |  |  |  |  |  |  |  |  |
| Carless & Douglas (2004) UK [33] |  |  |  |  |  |  |  |  |  |  |  |
| Carless & Douglas (2008a) UK [40] |  |  |  |  |  |  |  |  |  |  |  |
| Carless & Douglas (2008b) Location NR [39] |  |  |  |  |  |  |  |  |  |  |  |
| Carless & Douglas (2012) UK [32] |  |  |  |  |  |  |  |  |  |  |  |
| Carless & Sparkes (2008) UK [13] |  |  |  |  |  |  |  |  |  |  |  |
| Crone (2007) UK [41] |  |  |  |  |  |  |  |  |  |  |  |
| Evans (2017) UK [35] |  |  |  |  |  |  |  |  |  |  |  |
| Faulkner & Sparkes (1999) UK [38] |  |  |  |  |  |  |  |  |  |  |  |
| Graham et al (2017) Canada [31] |  |  |  |  |  |  |  |  |  |  |  |
| Hodgson et al (2011) Location NR [30] |  |  |  |  |  |  |  |  |  |  |  |
| Hoffman et al (2014) USA [34] |  |  |  |  |  |  |  |  |  |  |  |
| Irving et al (2003) UK [29] |  |  |  |  |  |  |  |  |  |  |  |
| Wärdig et al (2013) Sweden [28] |  |  |  |  |  |  |  |  |  |  |  |
| Yarborough et al (2016) USA [27] |  |  |  |  |  |  |  |  |  |  |  |
